# Food, fear and physics drive collective behaviour in the ocean's pelagic zone

**DOI:** 10.1098/rstb.2025.0208

**Published:** 2026-10-15

**Authors:** Kelly J. Benoit-Bird, William K. Oestreich

**Affiliations:** ^1^ Monterey Bay Aquarium Research Institute, Moss Landing, CA, USA; ^2^ Department of Evolutionary Biology & Environmental Studies, University of Zurich, Zürich, ZH, Switzerland

**Keywords:** pelagic, collective behaviour, schooling, diel vertical migration, group foraging, physical–biological interactions, social foraging, social evolution, ocean

## Abstract

Understanding how complex, coordinated patterns arise in animal groups has been an important theme in behavioural ecology, revealing much about the mechanisms of the evolution of behaviour. While fish schools are a well-known example, they represent just one case among numerous and diverse collective behaviours exhibited by pelagic animals, including schooling and swarming, vertical migration and cooperative foraging. We argue that key features of the pelagic ocean, including the limitation and patchy distribution of food resources, the lack of cover and the motion of the medium itself, have repeatedly led to the evolution of collective behaviour in ocean animals. Examples from animals as small as plankton and as large as whales, the surface and the deep and invertebrate and vertebrate taxa illustrate how fear, physics and food lead to the evolution of group behaviours. Collective behaviour itself also drives the development of group behaviour in other animals in the habitat. These feedbacks result in group behaviours that simultaneously serve multiple purposes, nested collective behaviours and multi-species collectives. Observations of life in the largest living space on Earth are providing new insights into the evolutionary drivers and adaptive significance of collective behaviour and group living.

This article is part of the theme issue ‘Multi-predator feeding aggregations in pelagic waters’.

## 1. Complex group behaviours from simple individual rules

Across the full range of organismal scales, from bacteria to whales, individuals form groups. Individuals in these groups influence the behaviour of other group members, profoundly affecting the ecological and evolutionary pressures on their neighbours [1]. Among the most visible outcomes of these interactions are organized, group-level patterns and movements. These apparently complex collective behaviours, including flocking in birds [2], swarming in insects [3] and migrating herds of mammals [4], can result from individuals following simple rules without central control, or a leader, of the group [3,5]. While generally less well-examined relative to terrestrial systems, collective behaviour occurs in a wide range of taxa in the waters of the ocean's pelagic ecosystems ( figure 1). Individuals in these collectives gain benefits such as protection from predators, proximity to mates and access to information, balanced by the costs of competing for resources with neighbours, increased attraction of predators and energy costs for coordination [6]. Here, we demonstrate that the widespread convergent evolution of collective behaviour in oceanic organisms arises from the challenges of and interactions between foraging, predation risk and physical forcing in the pelagic realm.

**Figure 1.**
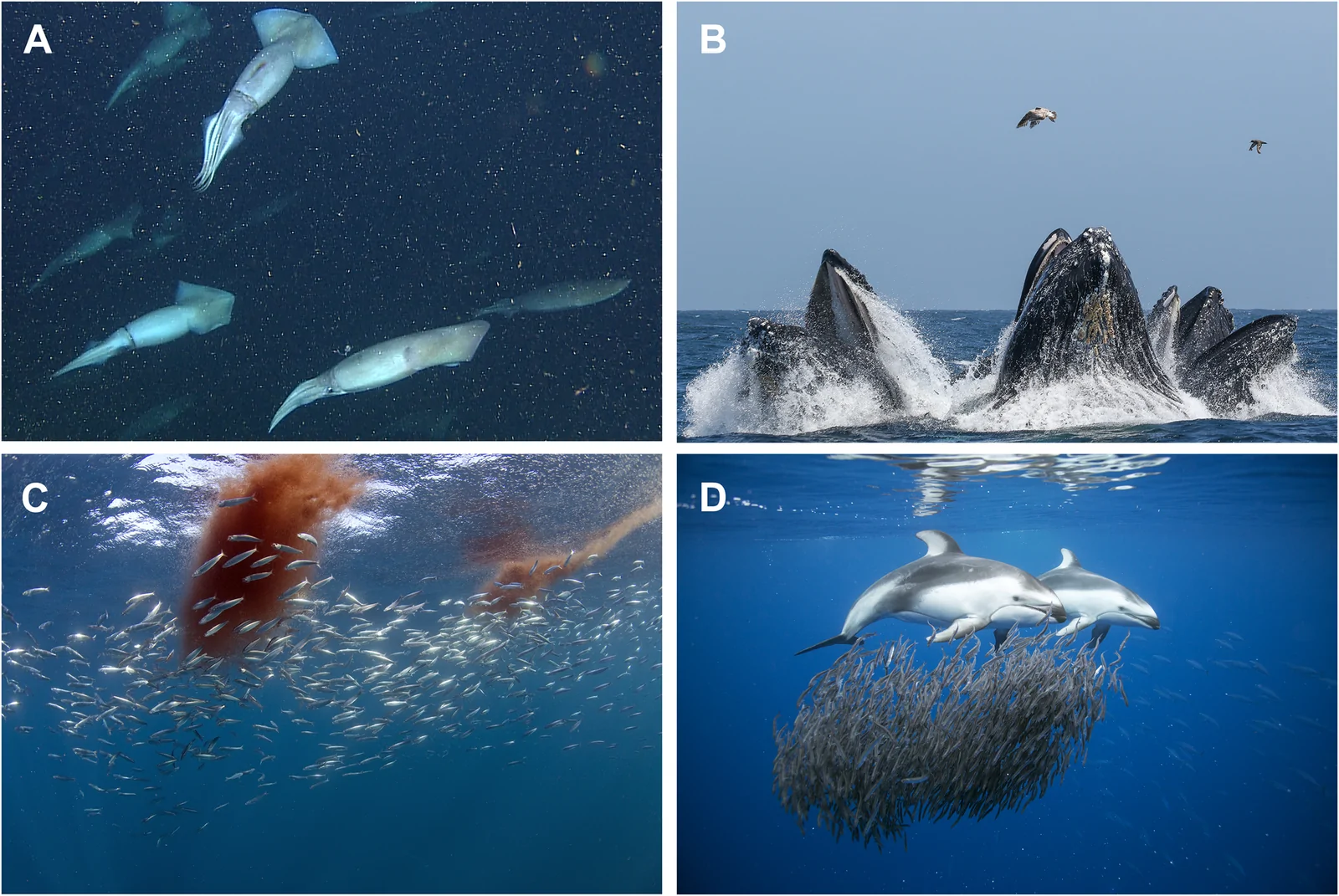
Examples of collective animal behaviour in the ocean's pelagic zone, the largest living space on our planet: (A) Humboldt squid hunting as a group on lanternfish after the fish migrated down into the twilight zone (Bruce Robison/Mbari); (B) humpback whales exhibiting coordinated lunge feeding on anchovies while seabirds take advantage of fish that escaped capture (Kate Spencer/Fast Raft); (C) a loosely packed sardine school feeding on a swarming krill (©Richard Herrmann/Minden Pictures); and (D) pacific white-sided dolphin pair-hunting tightly coordinated schooling anchovies (©Richard Herrmann/Minden Pictures).

## 2. Schooling by fish to balance the pressures of food, fear and physics

Collective behaviour is common in fish in pelagic habitats. The majority of marine fish species form social groups during at least part of their lifespan, while approximately one quarter are found in social groups throughout their life [7]. In fish, social groups of any nature are generally referred to as shoals, whereas organized, coordinated groups are called schools. Schools are most often comprised of individuals from a single size class of a single species that coordinate their movements to maintain relatively even spacing, polarization of heading and synchrony of turning and speed [8]. Extensive research has supported the benefits of schooling for reducing individual predation risk via increased predator detection (the ‘many eyes’ hypothesis), visual confusion of an attacking predator, dilution [9] and direct reduction in the attack rate of predators [10]. A combination of these mechanisms can be observed in the collective avoidance responses of schools under attack that include rapid compression or expansion of the school, splitting and merging of schools or the group rapidly circling behind the predator, creating a ‘fountain’ of shimmering bodies [9,11,12].

Social groups in fish have benefits beyond reducing individual predation risk. Grouping can increase spawning success by increasing access to mates and reducing predation on eggs [13]. Schooling can have direct foraging benefits, increasing individual feeding efficiency and the number of prey patches exploited [14]. The increase in sensory capacity and coordinated decision-making of the school important for avoiding predation also improves the amount and quality of information available to the group about other aspects of the environment [6]. For example, fish in schools are more efficient at climbing gradients in noisy environments like those typically found in the ocean [15]. Fish within schools are also more efficient in swimming. This reduces energy expended, increases access to patchy prey and can allow animals to maintain their position within their preferred habitats even when water motion is strong [16,17]. As these benefits change, fish dynamically modify their grouping behaviour, moving between loose shoal, coordinated school, and dramatic fountain of individuals [18]. Collective behaviour, including schooling, is a strategy that balances the ecological pressures faced by pelagic animals—access to food, predation and the physical environment they inhabit (figure 2).

**Figure 2.**
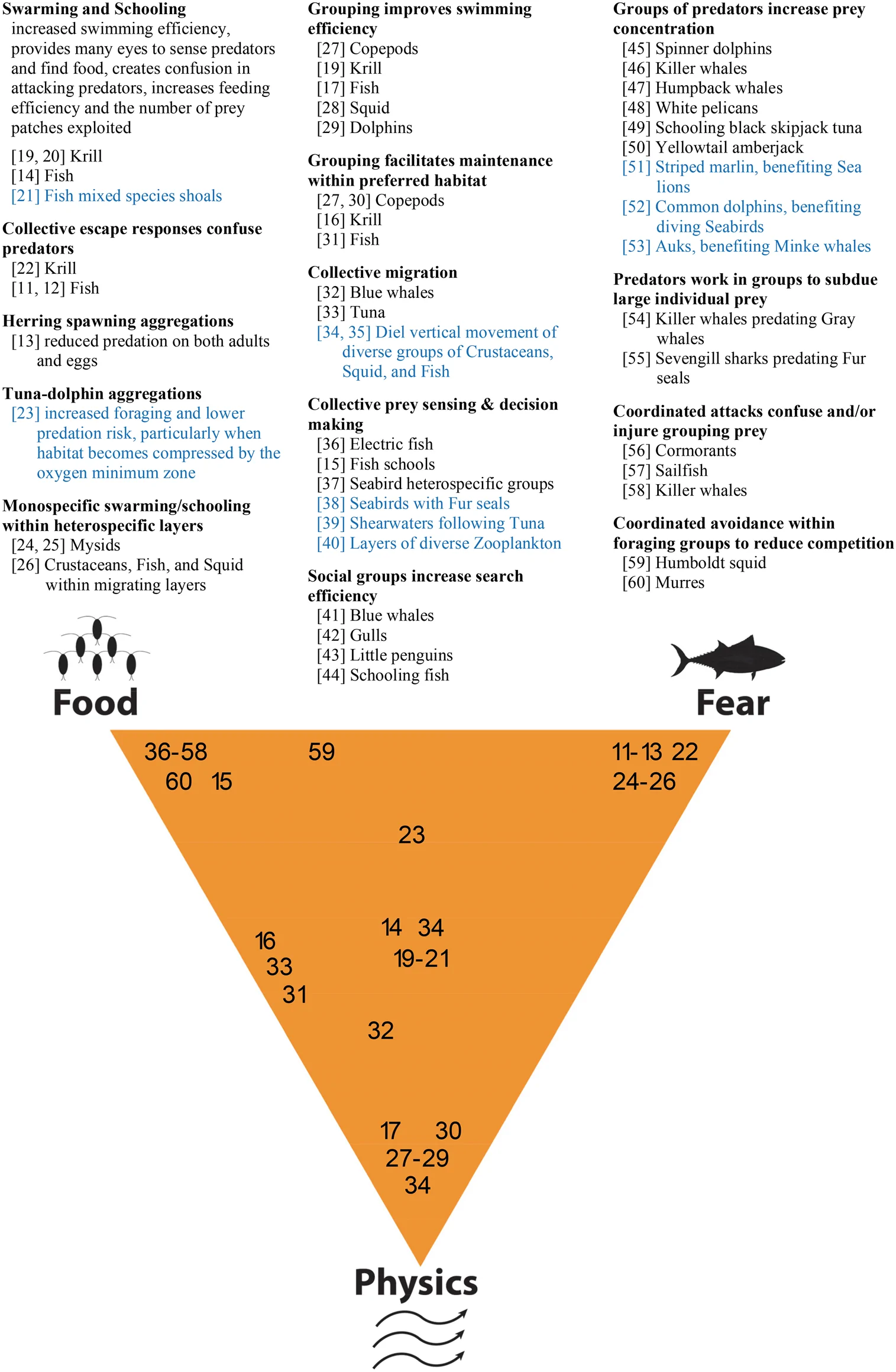
Food, fear and physics are three factors in the pelagic that mediate the ecology and evolution of collective behaviour. Selected examples of collective behaviour from a wide range of pelagic animals drawn from the literature [11–17,19–60] are plotted as a function of the relative importance of these factors. Each example is placed based on mechanisms identified in the cited publications, with multi-species groups shown in blue in the key.

## 3. The largest collective movement on the planet

The ocean's pelagic waters are home to Earth's largest example of collective behaviour. Throughout the world's ocean, crustaceans, squids and fishes form extensive layers. These aggregations move between the ocean's dimly lit interior where they hide from predators during the day and food-rich waters of the surface under the cover of nighttime's darkness [34], as illustrated in figure 3. Ten billion tons of small animals (0.1–30 cm) migrate each day [35]. This exceeds the biomass of the ‘Great Nile Migration’—a mixed-species annual migration that is thought to be the largest net animal movement on land—by at least several orders of magnitude.

**Figure 3.**
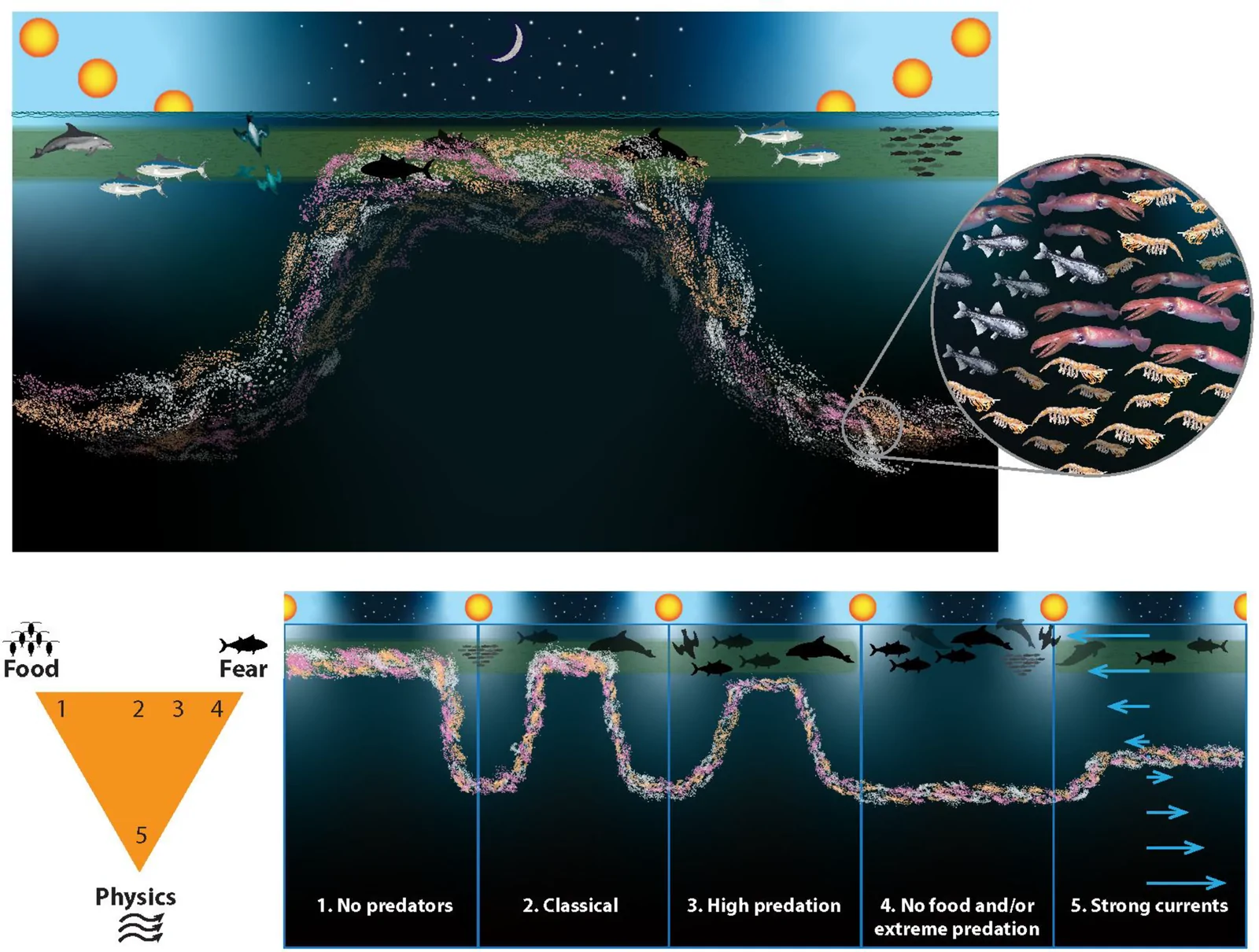
A diversity of vertebrate and invertebrate animals balance predation risk with access to food by migrating vertically between the dark depths of the ocean during the day and food-rich surface waters at night. As shown in the detail, within these migrating layers of animals, groups of similarly sized conspecifics form organized groups or schools in response to predation risk. These schools maintain contact with neighbouring groups to form extensive layers, nesting one collective behaviour within another. However, vertical migration is not a fixed pattern, as in the classical view, but is instead finely tuned to pressures imposed by food, fear and the physical environment. Examples of vertical migration patterns observed over a range of conditions are shown in the bottom panel. The relative importance of each pressure that the animals face changes through time. The relative strength of these factors is plotted on the orange multi-axis graph using numbers that correspond to each example illustrated. (Illustration by Mariah Salisbury/MBARI.)

At night, the collective of midwater animals is a multi-predator feeding aggregation. However, the individuals within the layers are also prey. The extent of animal migration, timing and even whether vertical movement happens at all are affected by the presence and behaviour of predators [61,62]. To continue to access food when predation risk is high, some migrating animals nest an additional form of collective behaviour within diel vertical migration. Monospecific, polarized groups or schools have been observed within multi-species layers [26,63]. Schools of squid respond to immediate predation risk by compressing and further synchronizing their behaviours, while neighbouring groups expand their spacing to maintain connection with the squid group, reflecting the importance of social behaviour both within and between species in these layers [26].

In addition to balancing food and fear, vertically migrating animals must cope with the risk of unintentional horizontal movement. Strong currents can quickly prove deadly, moving animals towards sloping seafloors where hungry mouths await, and away from productive habitats where food is abundant or into waters outside their physiological tolerances. To deal with strong flow, some species of small crustaceans in upwelling ecosystems spend time at their preferred depths in proportion to the relative onshore/offshore flow at each depth, allowing retention in food-rich nearshore habitats [30]. Small fish in these strong current systems modify their vertical migration behaviour when physical forces at their preferred depths exceed their swimming capacity, spending all day and night where currents are at a minimum, at the cost of reducing their access to food and exposing them to higher levels of predation (figure 3, example 5) [31]. Vertical migration is thus not just a fixed collective movement, but the result of highly dynamic collective decision-making that is tuned to fear, food and the physics of the pelagic environment.

## 4. Collective foraging is common in the pelagic

Social foraging, which is amongst the most successful life-history traits, occurs throughout the animal kingdom [64]. Foraging as part of a group provides many fitness benefits during the search for, pursuit and capture of prey [65] and is thought to promote the evolution of group living [66]. The most well-studied examples of groups pursuing and capturing prey are apex predators, particularly terrestrial carnivores [64]. In the ocean's open waters, examples of group hunting can be drawn from a wide range of taxa and locations within the food web, from millimetre-scale crustaceans to the largest marine mammals.

The benefits of foraging as part of a group often begin during the search for prey. By working in groups, foragers can increase both their search area and accuracy [36,67]. A greater range can be searched by groups as they travel using less energy and at greater speed than individuals, as shown in fish, crustaceans [14], squid [28] and dolphins [29]. With more searchers, efficiency in finding sparse prey increases, as shown in surface-feeding gulls [42], diving little penguins [43], schooling fish [44] and blue whales [41]. Social foraging groups also make more accurate decisions, effectively climbing noisy resource gradients by collectively averaging their sensory and decision errors [15].

Group foraging is also important during prey capture. As well-described in terrestrial carnivores, group foraging is particularly beneficial when capturing prey that are large relative to the predator—for example, killer whales predating grey whales [54] or sevengill sharks feeding on fur seals [55]. Group foraging can reduce the risks of injury to predators and increase their success in subduing prey. Important in pelagic systems, groups of predators also work together to keep the prey from sinking or drifting away while individuals feed [54].

Many predators in marine systems feed on prey much smaller than themselves [68]. To obtain the numerous small prey required, one successful strategy for pelagic predators is to work in groups to increase the concentration of resources. Herding behaviour has been observed in white pelicans [48], black skipjack tuna [49], yellowtail amberjack [50] and a variety of marine mammals (spinner dolphins [45], killer whales [46], humpback whales [47] and bottlenose dolphins [69]). Grouping is a common defence for animals of all sizes in the pelagic so some predators, including cormorants [56], sailfish [57] and killer whales [58], use coordinated attacks to confuse or even to injure prey, increasing their foraging efficiency.

One cost of group living is increased intraspecific competition. While foraging, some predators use coordinated avoidance to reduce those costs. For example, murres, a deep diving seabird, use social cues to find foraging habitats and then uniformly space themselves within these areas of elevated prey concentrations [60]. Similarly, Humboldt squid, which can exceed a metre in length, form groups made up of a single size class while foraging. Individuals in these groups carefully spiral around each other, actively avoiding feeding in the same patch of water, which results in the reduction of both competition and perhaps the risk of cannibalism that has been documented in this species [59].

## 5. Multi-species foraging groups occur throughout pelagic food webs

The pressures that drive collective foraging in the pelagic can also give rise to collective prey search and capture in groups comprised of heterospecific predators. In pelagic ecosystems, there are many examples with one predator species enabling access to prey for another. One commonly observed mechanism is one species driving prey closer to the surface and thereby allowing access to a second, dive-limited, air-breathing species. For example, some foraging whales use the surface to corral prey, incidentally bringing food into the diving range of seabirds [52]. Groups of predators concentrating small prey often benefit other species, such as common dolphins concentrating small fish and benefiting seabirds [52], or diving seabirds (auks) concentrating food that is taken advantage of by minke whales [53]. Some predators follow other predators while they hunt, taking advantage of the dead, stunned or scattered prey left behind after the primary predator begins their attack. This is an effective strategy, particularly for small predators like seabirds that are foraging on swarming or schooling prey and is frequently observed around whales that engulf large mouthfuls of these resources [53]. Many of these interactions may be commensal, with one species benefiting with little cost to the enabling individuals. However, there is some evidence for kleptoparasitism: for example, striped marlin find schooling prey and concentrate them to the surface before sea lions join in and interfere with the marlin's access to the food [51].

Heterospecific interactions can aid predators in finding prey. Local enhancement by heterospecifics has been shown to increase the knowledge of prey conditions in patchy environments for interacting seabirds and fur seals [38]. Shearwaters use tunas as cues to the presence of subsurface food that they cannot sense directly [39]. However, increases in search efficiency from multi-species groups are sometimes more explicit. Seabirds, for example, form groups with those with different foraging strategies and sensory capabilities while avoiding species within their own foraging guild [37]. Visual hunters pairing with chemosensory hunters increase the odds of finding prey while their different diving capabilities reduce interspecific competition—a model example of multi-species collective searching behaviour.

As in terrestrial ecosystems, most descriptions of multi-species predator groups in the pelagic are of predators at the highest trophic levels. However, the most ubiquitous examples of heterospecific foraging groups are found in mid-trophic predators. For example, layers of diverse zooplankton have been observed to collectively track layers of food resources [40]. Mysids emerge from the benthos to forage in single species swarms that form heterospecific layers [24,25]. And, while diel vertical migration has not been explored from this perspective, these abundant migrators represent the largest multi-species foraging group on Earth as these diverse assemblages migrate to the surface each night to forage [34,35]. Most of the predators in the ocean probably form groups not only to increase foraging success, but also to decrease predation risk. In the pelagic, collective behaviour often provides multiple fitness benefits simultaneously and must be viewed holistically rather than focusing on a single function. Examining the collective behaviour of the abundant mid-trophic level predators in pelagic systems promises to expand our understanding of the evolution of group living and the structuring of ecosystems.

## 6. Fear, food and physics

As illustrated in the examples highlighted here, collective behaviour is important for the survival of pelagic animals—from the smallest zooplankton to the largest whales. It is an adaptation observed in a diversity of taxa, including crustaceans, fishes, cephalopods and mammals. Why this widespread convergent evolution of collective behaviour in the pelagic?

The characteristics of the environment provide clues to answering this question. Much of the pelagic is remote and inaccessible to humans. When we catch glimpses beneath the surface of the waves, the blue seems to go on forever, interrupted only by an occasional scintillation of silver, a glinting mass of fish moving as one. The lack of cover available in the water column leaves no place for these fish to hide from predators, except amongst similar-looking neighbours [18]. For the fish, as well as their predators, food is sparse, patchily distributed and temporally ephemeral [70]. Because of the size structure of marine ecosystems, many predators are much larger than their prey [71] and must consume many individuals to survive, even when resource abundance is low. Many predators thus seek out aggregated prey [68], frequently as groups in order to overcome the collective defences of their prey [65]. The extremely aggregated distribution of resources and the size structure of marine food webs facilitate group foraging both within and between taxa [51,72].

The physics of the medium itself also play a key role in shaping collective behaviour in the ocean. Animals in the pelagic live in a viscous, cold fluid that is constantly in motion [70]. The cold water with its high heat capacity creates metabolic stresses, while viscosity makes swimming costly. Small animals must constantly move as momentum does not carry them forward and larger animals lose energy to drag. Simultaneously, the movement of the water itself carries animals away from their preferred habitats, which must be offset through intentional movement or avoidance of high flows. Collective behaviour is important in coping with these challenges. Organized, polarized swimming increases efficiency for animals of all sizes [17,19,27–29], while collective sensing and decision-making improve retention within preferred habitats [16,27,30,31].

These same physical attributes of the environment ensure that cues and signals from other animals—he social information that enables collective behaviour—abound in the pelagic. Movement through bioluminescent bacteria living on marine snow generates light [70], revealing individuals’ presence and behaviour in the darkness. Swimming generates flow, which is sensed by fishes’ lateral lines to maintain school cohesion [73]. Many cetaceans echolocate during prey pursuit and capture, producing conspicuous and widely propagating acoustic information about both consumer and resource distribution [74]. Keeping signals private in this environment is challenging, creating an abundance of social information that can provide knowledge about the environment beyond an individual's direct sensory capacity [75]. This social information is particularly valuable where resources are sparse, patchy and ephemeral, leading to collective sensing and decision-making among conspecifics—as observed in the coordinated onset of seasonal migration in blue whales [32,41] or the group hunting in spinner dolphins [45,76]. Although such social signals are intended for conspecifics, they also represent valuable social information for eavesdropping heterospecifics, encoding information about biophysical ecosystem variation [77] and facilitating multi-predator foraging aggregations [78]. The abundance of social information and its value in the pelagic create high potential for the emergence of collective behaviour across taxa and scales.

## 7. Plus feedback

In order to survive, animals in the ocean must find sparse, patchy food while avoiding predators within a constantly moving environment lacking refugia. Collective behaviours provide a number of solutions to this combination of challenges. As individuals balance fear, food and physics, their reasons for forming groups change and any one behaviour may present multiple advantages and trade-offs. This balancing can lead animals to exhibit collective behaviours that are nested within larger-scale collective patterns, each with specific benefits [24–26]. The behaviours of conspecifics, competitors and predators can also influence grouping, with collective behaviour in one animal influencing the development of group behaviours in another in a feedback loop. For example, schooling in fish drove the development of group foraging in many of their predators [65]—an arms race of sorts. Similarly, diel vertical migration of small zooplankton instigated the migration of planktivores that drove migration in their predators, and so on—a collective behaviour that cascaded through trophic levels [79]. The commonality of collective behaviour across sizes and taxa in the pelagic sets up opportunities for multiple-species aggregations, including multiple predator foraging groups, as diverse animals seek to solve similar problems, sometimes in complementary ways.

## 8. The ocean provides insights into collective behaviour across Earth

The convergent evolution of collective behaviour across diverse pelagic taxa indicates that the oceanic realm is a rich space for enhancing breadth and depth in understanding the ecology and evolution of collective behaviour. The unique biophysical characteristics of this environment, including the limitation and patchy distribution of food resources, the lack of cover and the motion of the medium itself, represent an end-member on the spectrum of Earth's biomes. Aerial systems are also vast, fluid, three-dimensional, yet its inhabitants are also tied to the distinct pressures of life in the terrestrial realm at the other end of the biophysical spectrum. A lack of practical observational capacity has historically limited detailed and persistent observations of pelagic organisms’ behaviour, thus impeding rigorous hypothesis testing both within pelagic ecosystems an in comparison with other biomes. This situation is rapidly shifting with the integration of traditional and modern techniques. Detailed observations are increasingly possible via active acoustic sensing [80], spatially explicit passive acoustic sensing [81], underwater imaging systems [82] and bio-logging devices equipped with auxiliary sensors and tailored to an increasing diversity of taxa [83]. These methodological advances, many of which can be integrated with autonomous platforms to enhance observational continuity and/or duration, provide an unprecedented window into pelagic animals’ lives, promising insights about the drivers and dynamics of their collective behaviour. We highlight four areas of focus in pelagic ecosystems that promise particular gains for our understanding of collective behaviour (Box 1): consideration of neighbouring taxa as a force driving collectives, observation of individual mid-trophic animals, untangling the influences of physical forcing and examination of the sensory capabilities of animals in mixed-species groups.

**Box 1: Priority areas for pelagic collective behaviours**

**Consideration of neighbours as drivers of collective behaviours:** group behaviours may be nested and often serve multiple functions simultaneously. All of the drivers and benefits of collective behaviour need to be examined simultaneously, with neighbouring taxa deserving special attention. We hypothesize that collective behaviours are more likely to evolve when organisms with which a species interacts exhibit collective behaviour. For example, even modest gains in predation avoidance by groups should increase the relative risk of being alone, creating pressure for grouping to reduce predation risk in competing species. Similarly, predators that rely on grouping or aggregated prey are more likely to exhibit collective or group hunting than predators relying on solitary resources.

**Observation of individual mid-trophic animals:** only rarely has the behaviour of *individual* mid-trophic animals within groups been examined. We predict that mid-trophic species are most likely to inhabit the middle of the fear, food and physics forcing triangle, balancing all three forces in making individual decisions that lead to collective behaviour. The creative application and integration of tools will be necessary in these smaller-bodied species but will provide the opportunity to examine dynamic responses to diverse biophysical pressures.

**Careful attention to physical forcing:** the contribution of physical forcing to the evolution of group behaviour has probably been underappreciated as it may often covary with predation risk—both probably decrease with increased swimming ability/size. The effects of fear and physics on behaviour may be synergistic or oppositional and must be untangled carefully.

**Examination of sensory capabilities in mixed-species groups:** comparative analysis across taxa and systems can shed light on the drivers of groups, with multi-species groups providing an opportunity for simultaneous comparison within a specific set of conditions. Sensory differences are one variable that should be carefully characterized. We predict that multi-species foraging groups are likely to be more common when their member species have diverse sensing modalities (e.g. visual versus acoustic versus chemosensory hunting) and/or scales (e.g. fine- versus broad-scale search capacities).

New discoveries in ocean animal behaviour carry significance not only for pelagic ecology, but also for our theoretical understanding of collective behaviour across Earth's ecosystems that may extend to collective processes in cell biology, neuroscience, robotics and economics [5]. Comparative studies and theoretical development in collective behaviour must account for the breadth of ecosystem conditions in which collective behaviour has evolved on Earth. Pelagic ecosystems represent the majority of Earth's biosphere. With their unique biophysical conditions and the diverse collective behaviours spread widely through the food web, pelagic ecosystems represent a critical space for developing deeper understanding of the ecology and evolution of collective behaviour.

## Data Availability

This article has no additional data.

## Theme

One contribution of 14 to a theme issue ‘Multi-predator feeding aggregations in pelagic waters’.
